# Data on post bank customer reviews from web

**DOI:** 10.1016/j.dib.2020.106152

**Published:** 2020-08-08

**Authors:** Andrei Plotnikov, Alexey Shcheludyakov, Vadim Cherdantsev, Alexey Bochkarev, Igor Zagoruiko

**Affiliations:** a Perm National Research Polytechnic University, 29, Komsomolsky *Av*. 614990, Perm, Russian Federation; b Perm State Agro-Technological University named after Academician D.N. Pryanishnikov, 23, Petropavlovskaia St., 614990, Perm, Russian Federation; c ^c^Perm State National Research University, 15, Bukireva st., 614990, Perm, Russian Federation

**Keywords:** Data mining, Text mining, Reputation management, Online behaviour, Client satisfaction

## Abstract

This document describes a set of customer feedback data concerning the Post Bank. We collected data from 16,659 feedback lines using the Beautiful Soup package from the authoritative site banki.ru is selected as the source of data for collection. The dataset is compiled to monitor the level of trust of bank customers in its banking service. The data presents text reviews for 2013 - 2019 and includes, with or without ratings. Scientists can predict feedback ratings with an empty value in the future. We added additional columns to the dataset with official comments of bank employees, as well as values for the fog-index by Gunning parameter, which is used for the readability of the text. The data can be useful for customer service managers to identify problems in customer service and solve these problems, to assess the dynamics of the appearance of positive and negative reviews of bank customers.

**Specifications Table**

**Table:** 

**Subject**	Business, Management and Accounting (General); Marketing
**Specific subject area**	The data collected relates to the field of reputation management in the banking sector of the economy. Customer feedback is a reflection of banking services and contains reasons for both positive and negative.
**Type of data**	Table, xlsx
**How data were acquired**	We used Data mining techniques – Beautiful Soup is a Python programming language package for parsing HTML documents.
**Data format**	Raw, Analyzed Raw data collected by using the Beautiful Soup package from the authoritative site banki.ru Table columns such as ‘response_message_length’, ‘gunning_fog_index’, ‘binary_score’ in the xlsx file contain analyzed data.
**Parameters for data collection**	The dataset represents collection customer feedback published from 2013 to 2019 in the Russian leading finance website www.banki.ru[1]. Data was groped during November and December 2020 the collection process in one table from each item observations. Data has additional columns with Gunning fog-index parameters.
**Description of data collection**	We have collected data from open sources. Many banks offer a similar set of services for approximately the same price in a competitive market. Reputation becomes more critical when formed on the Internet-based on Web 2.0 technology (User-generated content). Data contains customer feedback text, responses rating grade (1, …, 5; 1 is the most negative, and 5 is the most positive), responses header, responses DateTime. Size: 16,659 items.
**Data source location**	Russia
**Data accessibility**	Repository name: Mendeley data Data identification number: DOI: 10.17632/rfkh49b6s5.3 Direct URL to data: https://data.mendeley.com/datasets/rfkh49b6s5/3

**Value of the Data**

• The dataset is very interesting for data analysts, heads of banking departments for evaluating customer reviews by responses header and messages. Responses header is title of review/ Response header is title of review.
• The data set is presented in a systematic table for the convenience of subsequent data analysis.
• Data can be useful for educational purposes for students of data analysis and machine learning algorithms.
• Future researchers can use the data set to identify trends for positive and negative reviews and to identify the causes of negativity and positives by messages.
• Managers can use the data to evaluate customer service objectively.
• Managers can change business processes based on a set of feedback data. As users indicate in their reviews, significant advantages and disadvantages for them.

## 1 Data description

The dataset (see xlsx (Excel) file with this article) contains collected customer reviews of Post Bank. For the convenience of describing each column, refer to Table 1, which includes the column name, the number of filled values in the column, and data type. Information from the dataset (xlsx) makes it possible to group or create filters to analyze this data by columns.

**Table 1:** Columns data type.

No	Column	Count	Data type
1	responses_header	16,659	object
2	responses_rating_grade	11,222	float64
3	responses_status	7559	object
4	responses_message	16,659	object
5	responses_datetime	16,659	datetime64[ns]
6	reviewer	16,659	object
7	comments	16,659	int64
8	views	16,659	int64
9	id	16,659	int64
10	have_email	16,659	int64
11	responses_message_length	16,659	int64
12	gunning_fog_index	16,659	float64
13	binary_score	16,659	object

Table 1 describes variables in columns of Excel spreadsheets and data type of variables:

1. ‘responses_header’ can take any value. Response header is title of review (message);
2. ‘responses_rating_grade’ takes values from 1 (negative) to 5 (positive);
3. ‘responses_status’ can take two values: Problem solved (if the bank solved the client's problem) / Not counted (Evaluation with this status does not affect the Bank's People's Rating), checked (status is not permanent), empty value (the review passed the test and affected the bank rating);
4. ‘responses_message’ is a response from an official representative of the bank;
5. ‘responses_datetime’ is the time and date of the left review;
6. ‘reviewer’ is a nickname or private email;
7. ‘comments’ is the number of comments to recall;
8. ‘views’ are the number of views;
9. ‘id’ is a unique recall identifier;
10. ‘have_email’ is the presence of an email if the user has not registered as a user with a nickname;
11. ‘responses_message_length’ is the number of letters in the response message;
12. ‘gunning_fog_index’ is the value of text perception assessment [2], - how much the text is readable and devoid of “incomprehensibility”;
13. ‘binary_score’ is a review rating.

Data types in Table 1:

• object is text data;
• float64 is floating point number;
• datetime64[ns] is date in YYYY-MM-DD format and time;
• int64 is an integer.

**Fig. 1:**
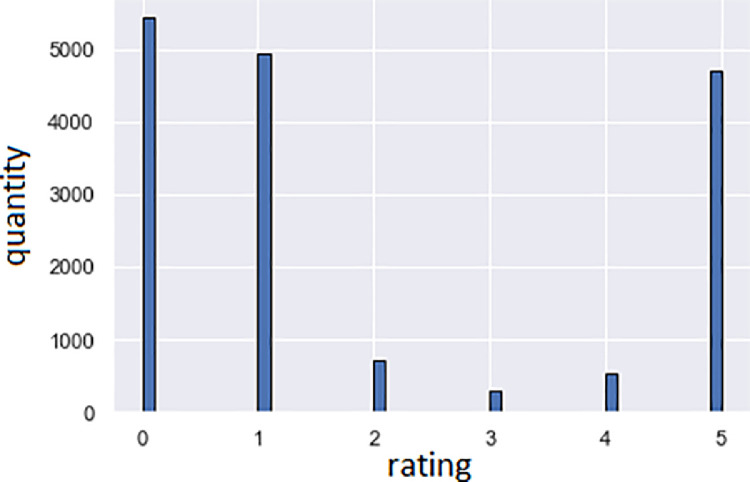
Responses rating distribution.

Fig. 1 shows us client feedback rating distribution. The X-axis shows the feedback rating; Y-axis shows the number of reviews. Legend: 0 - not rated; 1, …, 5, where one is the most negative, and 5 is the most positive assessment.

Descriptive statistics is engaged in the processing of empirical data (‘reviewer’ column in xlsx file), its systematization, visual representation in the form of graphs and tables, as well as their quantitative description through the leading statistical indicators. Number of reviews from one user: minimum number of reviews: 1; the maximum number of reviews: 8; the average arithmetic number of reviews: 1.1; fashion, the number of reviews: 1.

**Fig. 2:**
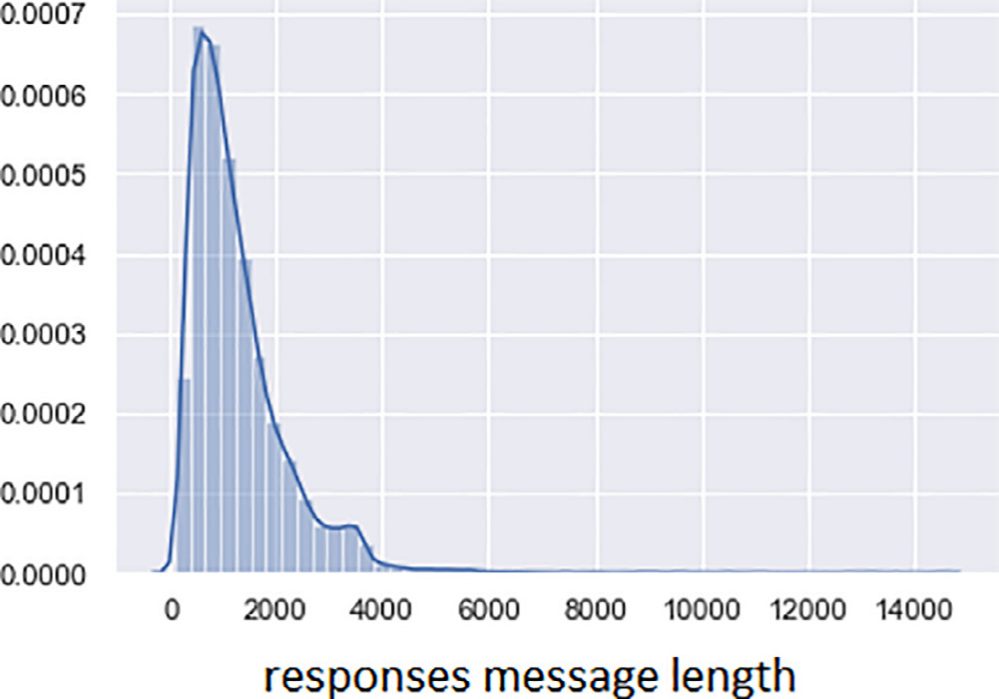
The distribution of the length of the texts of reviews.

The X-axis shows the number of characters; The Y-axis shows the share in the total volume of all reviews in Fig. 2. Average responses message length is 1263 characters; The standard error is 7.2; The median is 1017; The standard deviation is 922.8; The shortest review contains 80 characters and the most extended 14,465 characters. The most significant number of reviews contains up to 2000 characters. Fig. 2 and the description are presented based on the calculations on the responses_message_length column of the table (see Excel file with this article).

**Fig. 3:**
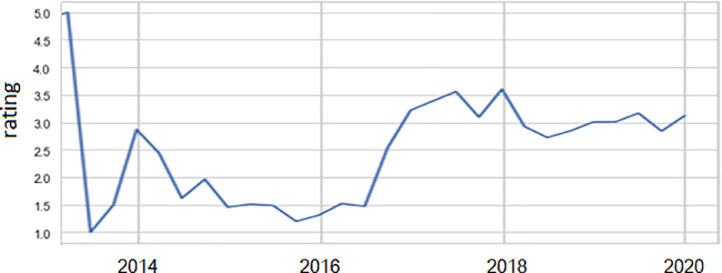
Changing responses rating depending on responses_datetime.

Fig. 3 illustrates us the change in average responses rating depending on responses DateTime and seasons.

In the second half of 2013, the first reviews appeared with the highest possible rating, and the average rating went into decline. We observe that in the second half of 2016, the average rating of reviews began to increase. Fig. 3, and figure description are presented based on the calculations on the responses_datetime column of the table (see Excel file with this article).

**Fig. 4:**
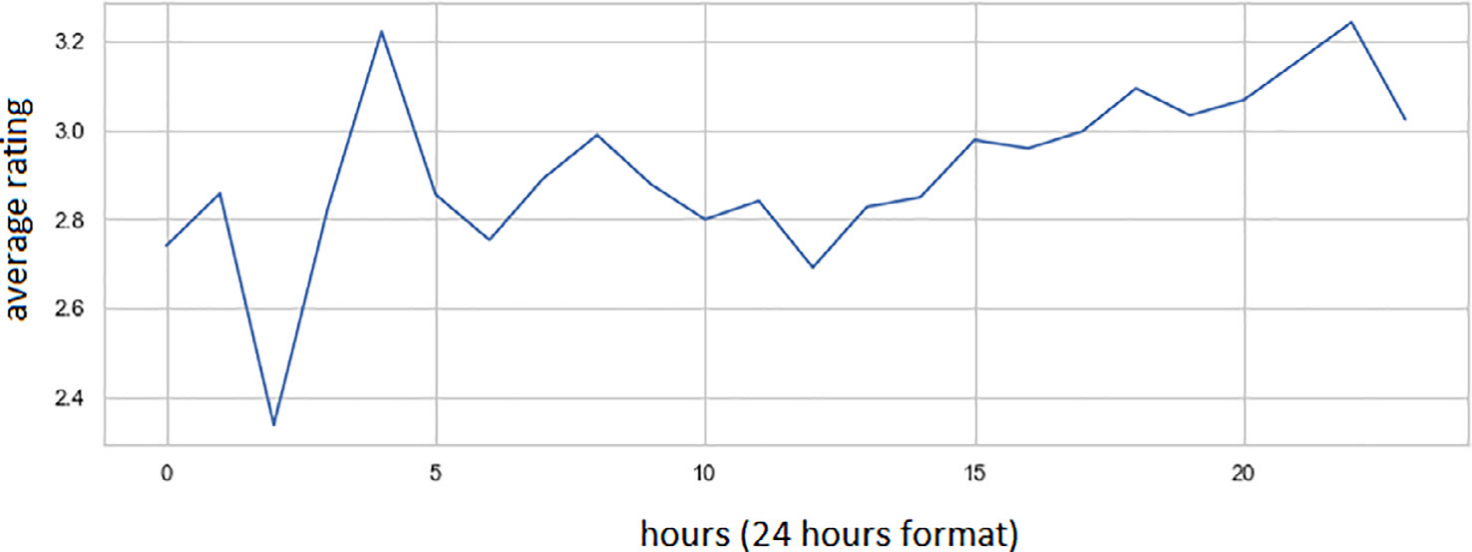
The dependence of the average response rating on the time of day.

Fig. 4 shows us the dependence of the average score on the time of day. We determined the time of day based on the responses_datetime column (see Excel file with this article).

Table 2 shows the correspondence of the average length of reviews concerning the binary classification. Responses rating equals ‘1′ or ‘2′ are negative, and rating equals ‘4′ or ‘5′ are positive. We abstained from giving a binary rating of Responses rating equals ‘3′. We determined these values based on the ‘binary_score’ column depends on ‘responses_message_length’ column (see Excel file with this article).

**Table 2:** Comparison of the lengths of positive and negative reviews.

binary_score	responses_message_length
**negative**	1474.2
**positive**	1041.6

**Fig. 5:**
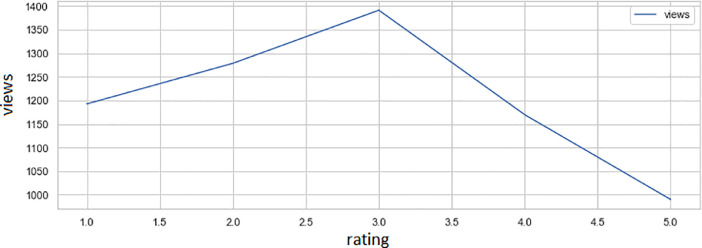
Distribution of the average number of views from rating grade.

Table 3 and Fig. 4 shows the dependence of the average number of views (Y-vertical axis on Fig. 4) on rating grade (X-horizontal axis on Fig. 4). The most significant number of observations belongs to the three rating grades, and perhaps this is because the estimation uncertainty (three is closer to rating bad or good?).

**Table 3:** The number of views depending on the rating.

responses_rating_grade	views
**1.0**	1192.8
**2.0**	1278.9
**3.0**	1391.4
**4.0**	1169.8
**5.0**	990

**Fig. 6:**
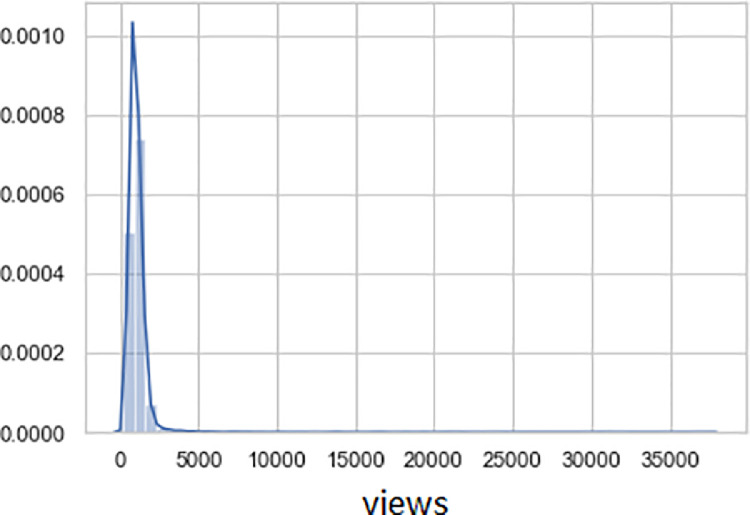
Distribution of views by reviews.

**Fig. 7:**
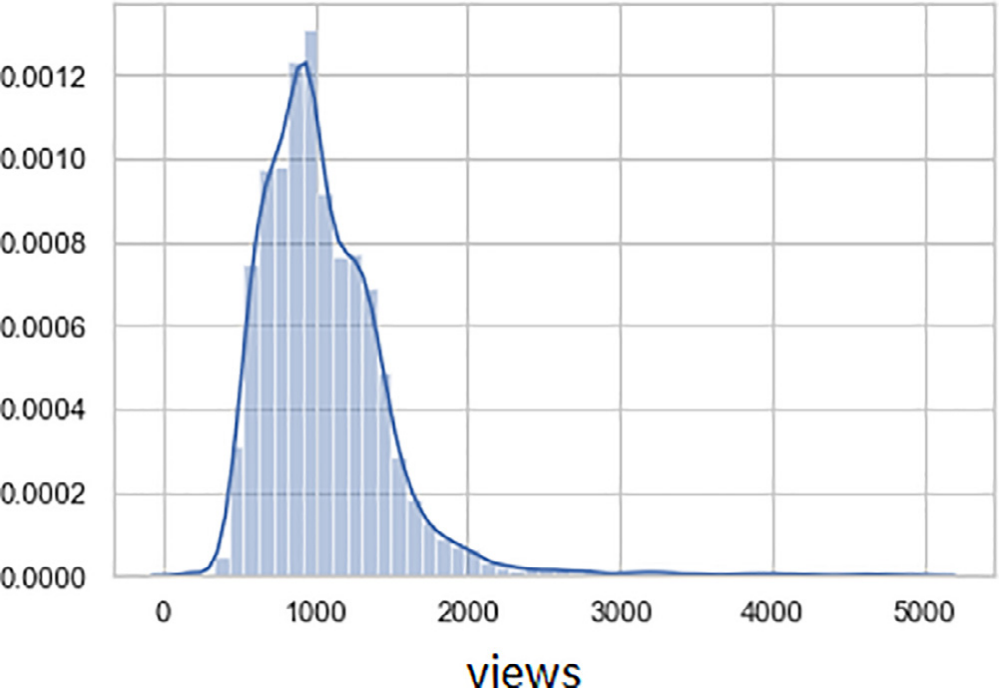
The distribution of the number of views by reviews (with a limit of 5000 views).

The horizontal X-axis illustrates the distribution of the number of views, and the vertical Y-axis shows the proportion of views in the total dataset in Fig. 6, Fig. 7. Also, Fig. 6 shows us the lengthened tail distribution of the number of views. The average number of review views is 1.150; The standard error is 8.3; The median number of views is 992; the standard deviation is 1072.8; the minimum amount of views is 139, and the maximum is 37,471.

## 2 Experimental design, materials, and methods

We built pivot tables using the Pandas package; using the matplotlib package, we plotted the graphs for the obtained datasets. We designed the charts using the seaborn package. [3] We used descriptive statistics is engaged in the processing of empirical dataset.

### 2.1 Gunning fog-index

The readability of the text is an index indicating the effort that the reader or listener needs to understand the essence of the text. [2]

When using the Gunning fog-index, it should be borne in mind that the calculation method was originally developed for the analysis of texts in English. When adapting the formulas used, it is necessary to take into account the specifics of a particular language in which the studied text datasets are written. So, English is much more informative than Russian. The difference in the text volume with the same semantic load is, on average, 20%. When translating a text from Russian into English, the expressions are much shorter, and when translating from English into Russian - vice versa. [6]

The column with the heading ‘gunning_fog_index’ in xlsx file with the dataset contains values with a correction factor is 0.78 for the text analysis [4]. Gunning fog-index was calculated in a modified Textstat package [5] in a Python programming language. We added a correction factor = 0.78 for text in Russian because the original Textstat package is useful for English text only. I. Oborneva [6] and T. Litvinova et al. [4] explain the value of the coefficient in their paper in relation to the Russian language, and we will not focus our attention on it in this data article. Thus, we use the formula with the correction factor:

$$Fog\;index=0.4[0.78\left(\frac{words}{sentences}+100\left(\frac{complex\;words}{words}\right)\right]$$

“Word” has one or two syllables; “complex word” has three and more syllables.

The range of values: from 70 and above - no specialized training is required; up to 70 - secondary education; up to 60 - the intellectual level of training; up to 30 - for understanding, you need a scientific degree of training. These values can later be used to evaluate customers.

### 2.2 Correlation matrix

**Fig. 8:**
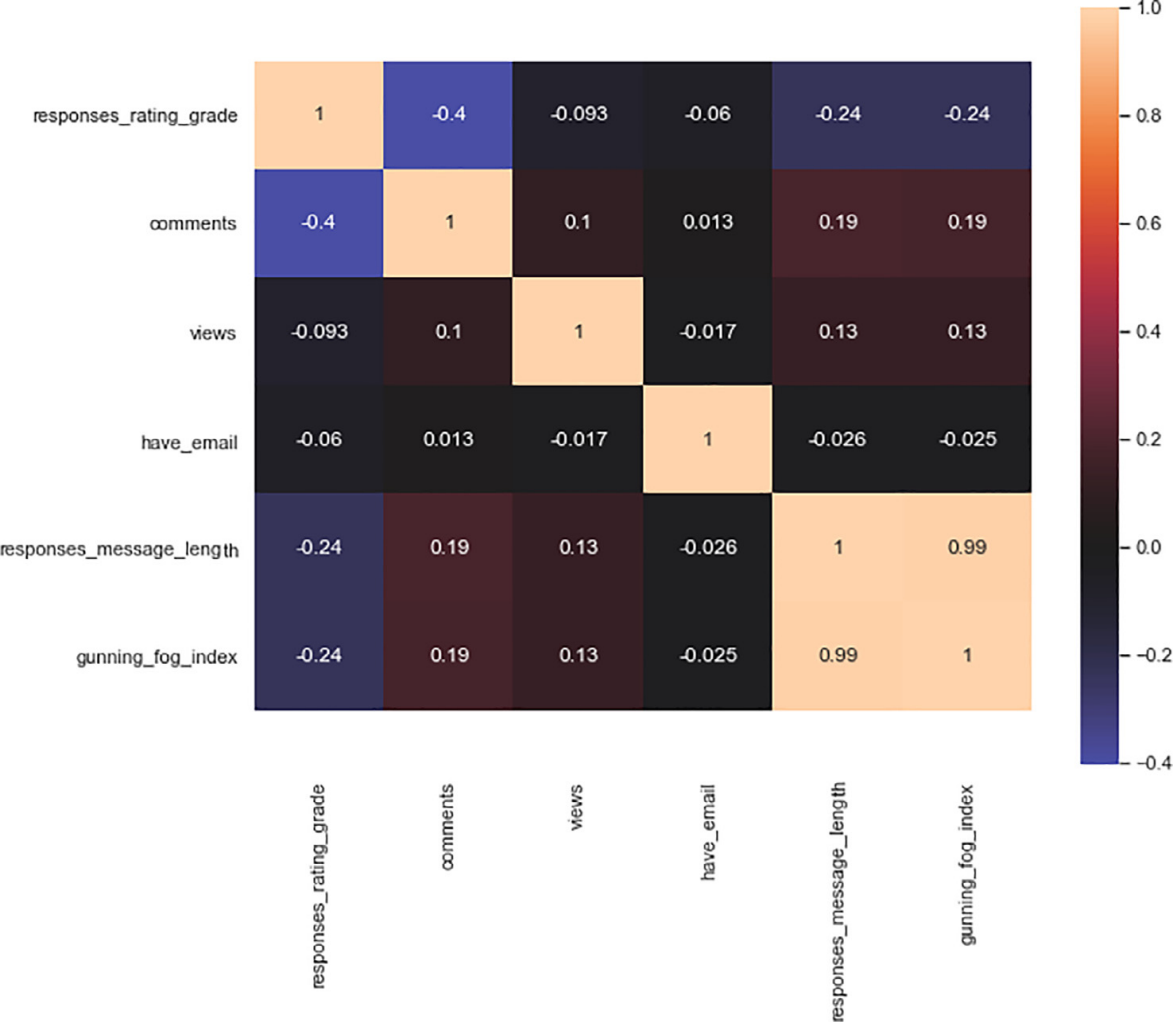
The correlation matrix.

As a result, we will create a correlation matrix in all respects and display it graphically on Fig. 8.

The correlation matrix on Fig. 8 is a square table in which at the intersection of the corresponding row and column there is a correlation coefficient between the corresponding attributes (columns in Excel file in this article): 'responses_rating_grade', 'comments',' views', 'have_email', ‘responses_message_length', 'gunning_fog_index'. The color shows us the power of interconnection. We observe an inverse correlation between the two variables, 'comments' and 'responses_rating_grade' (−0.4). The inverse correlation has between the two variables so that they move in opposite directions has moderate significance. When 'responses_rating_grade' decreases, the number of 'comments' increases. Note that there is an inverse correlation with a low significance between the variables' responses_rating_grade 'and' gunning_fog_index '(−0.24), as well as an inverse correlation with a low significance between the variables' responses_rating_grade' and 'responses_message_length '(−0.24). A strong correlation direct correlation (0.99) was found between the two variables' gunning_fog_index 'and ‘responses_message_length'.

## Ethical statement

Before data was collected from the Website, we researched the User Agreement (https://www.banki.ru/rules) The agreement contains paragraph 7.4, which declares the following: All Banki.ru website materials can be reproduced in any media, on Internet servers, or any other media without any restrictions on the volume and timing of publication. This permission applies equally to newspapers, magazines, radio stations, television channels, websites, and Internet pages. The only condition for reprinting and relaying is a direct link to the source https://www.banki.ru. Prior Consent for a reprint by publishers or authors of the Website is not required. It means that we can use any amount of data by referring to the source.

We have not violated anyone's interests in the data article. We treat the employees of the bank, shareholders, and customers with respect.

Appendix A. Supplementary data

Supplementary data to this article can be found online at

http://dx.doi.org/10.17632/rfkh49b6s5.3

## Declaration of Competing Interest

The authors declare that they have no known competing financial interests or personal relationships which have, or could be perceived to have, influenced the work reported in this article.

## Appendix Supplementary materials

Image, application 1
